# Otolithic organs and vibratory sensitivity in frogs: do saccular otoconial volumes vary according to lifestyle?

**DOI:** 10.1007/s00359-026-01826-0

**Published:** 2026-07-21

**Authors:** Matthew J. Mason, Sandra Goutte, Piers J. Seal, Marius Kluonis, Jakob Christensen-Dalsgaard

**Affiliations:** 1 Department of Physiology, Development & Neuroscience, University of Cambridge, Downing Street, Cambridge, CB2 3EG UK; 2 Division of Sciences, New York University Abu Dhabi, PO Box 129188, Abu Dhabi, United Arab Emirates; 3 Department of Natural History, NTNU University Museum, Trondheim, Norway; 4 Department of Biology, University of Southern, Campusvej 55, Odense M, DK-5230 Denmark

**Keywords:** Frog, Otolith, Sacculus, Seismic sensitivity, Fossorial

## Abstract

Of the three otolithic organs found in frogs, the sacculus is known to possess acute sensitivity to seismic vibrations. Simple models of otolith function suggest that increasing size of an otolith should improve vibratory sensitivity. In the first part of this paper, we provide a short review of the existing literature pertaining to the vibratory sensitivity of the otolithic organs in frogs. Given parallels in other vertebrate groups, we hypothesized that fossorial frogs would have larger saccular otoconial masses (‘otoliths’) than non-burrowing species, used for the detection of ground vibrations. In the second part of this paper, we tested this hypothesis by measuring otolith volumes in 15 species of frogs, based on CT reconstructions. Larger species tended to have larger otoliths. The fossorial frog *Hemisus guineensis* had the largest otoliths relative to skull size of all species examined, followed by a non-fossorial species, *Ptychadena delphina*. The otoliths of two other fossorial specialists were not notably enlarged, however, and volumes were relatively small in some semi-fossorial species. Pronounced sexual dimorphism was noted in the aquatic frog *Xenopus laevis*, in which males have larger saccular otoliths than females in absolute terms, despite their smaller body size. Otoconial dissolution represents a problem for studies which involve the examination of preserved specimens, but with this caveat in mind, we conclude that factors other than fossoriality might drive expansion of the saccular otoliths in frogs.

**Supplementary Information:**

The online version contains supplementary material available at https://doi.org/10.1007/s00359-026-01826-0.

## Introduction

This paper was written in tribute to the work of Prof. Peter Narins. A pioneer in the field, Peter Narins demonstrated vibratory sensitivity in several species of frogs from both electrophysiological and behavioural perspectives, focusing on the role of the sacculus. He also made important advances in the study of seismic sensitivity in burrowing mammals (e.g. Narins et al. 1997). Two of the current authors (JCD, MJM) had the privilege of working in the Narins laboratory at UCLA on the sensory abilities of frogs. We have both benefited from Peter Narins’ excellence in fieldwork, and his combination of interest in natural history and animal behaviour with rigorous scientific experimentation.

There are two parts to this paper. We begin with a review of the literature relating to the otolithic organs of frogs and their role in detecting vibrations, drawing on the seminal work performed by Peter Narins and his collaborators. In the second part of the paper, we address the question of whether saccular otoliths are enlarged in fossorial frogs, so as to augment seismic sensitivity. We also examine volumes of the saccular otoliths in the aquatic frog, *Xenopus laevis*, and demonstrate sexual dimorphism in this species. We believe that this is the first study to compare otolith volumes in adult frogs, in a quantitative way.

### Part 1: literature review

The frog inner ear contains a number of different sensory organs, with the three semicircular canals and the utriculus in the upper part (pars superior) of the membranous labyrinth and the sacculus, lagena and auditory papillae in the lower part (pars inferior). Across vertebrates in general, the pars inferior shows the greatest variability, while the pars superior is similar in all gnathostomes (Retzius 1881, 1884). The evolutionary development of the sensory organs of the ear in vertebrates is considered by Fritzsch et al. (2002).

**Fig. 1:**
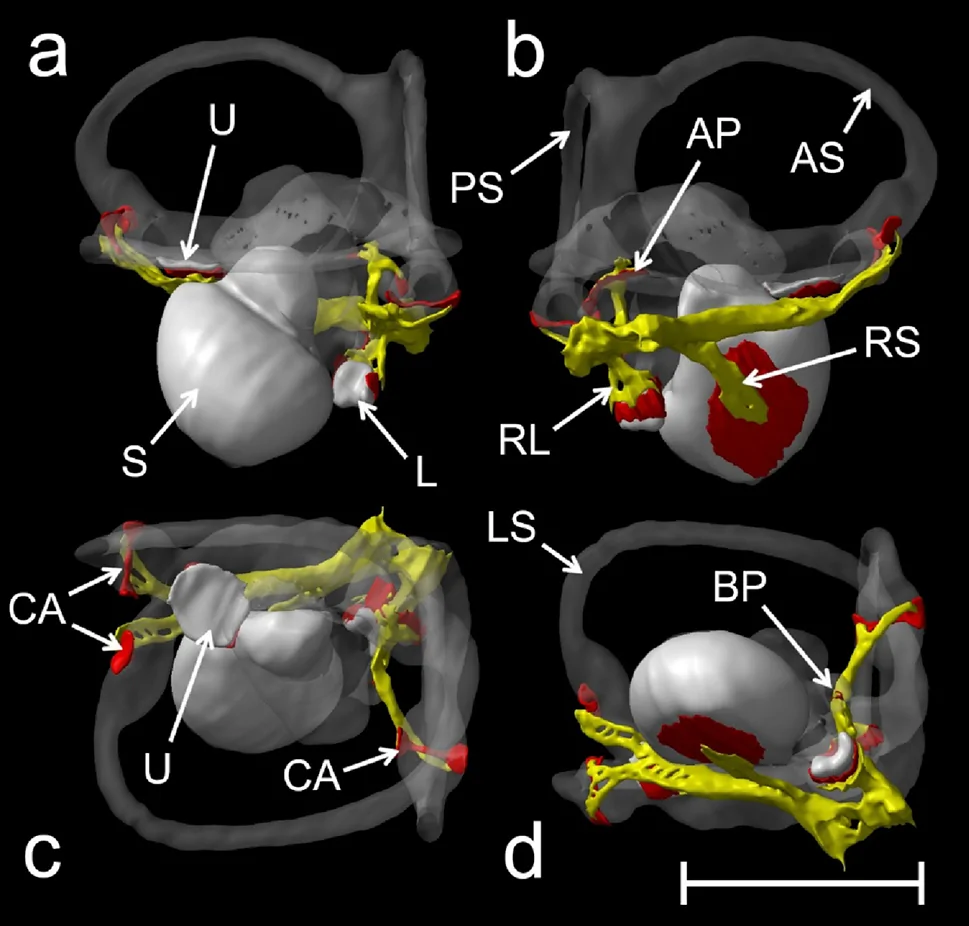
Reconstructions of left inner ear structures of the frog *Lithobates pipiens*, seen from approximately **a** posterolaterally, **b** anteromedially, **c** dorsally and **d** ventrally. The endolymph-filled otic labyrinth (translucent grey), sensory nerve branches (yellow) and sensory epithelia (red) were reconstructed using Stradview 7.321, based on serial sections obtained as part of a previous study (Mason et al. 2015). The lagenar (L), saccular (S) and utricular (U) otoliths were not visible in the sections owing to dissolution; their shapes and positions were estimated based on containing structures and CT scans of the same species, also described in Mason et al. (2015). They are shown in white. AP = amphibian papilla; AS = anterior semicircular canal; BP = basilar papilla; CA = crista ampullaris; LS = lateral semicircular canal; PS = posterior semicircular canal; RL = ramus lagenae; RS = ramus sacculi (saccular nerve). Scale bar 2.5 mm

Of the three otolithic organs, sacculus, utriculus and lagena, the sacculus is the largest in frogs (Fig. 1). Each features a sensory macula containing hair cells, which are coupled to a mass of otoconial crystals. The endolymphatic sac, which enters the cranial cavity and extends down the vertebral canal (Whiteside 1922), also contains otoconia. The endolymphatic sac otoconia appear to act as a calcium store and pH buffer, rather than having any sensory function (Pilkington and Simkiss 1966; Simkiss 1968). Amphibian otoconial crystals are formed from calcium carbonate; the saccular, lagenar and endolymphatic sac crystals are aragonite while the utricular crystals are calcite (Marmo et al. 1983a; Kido and Takahashi 1997). The density of aragonite, 2.94 g cm^− 3^, is slightly greater than that of calcite, 2.75 g cm^− 3^ (Bragg 1924).

**Fig. 2:**
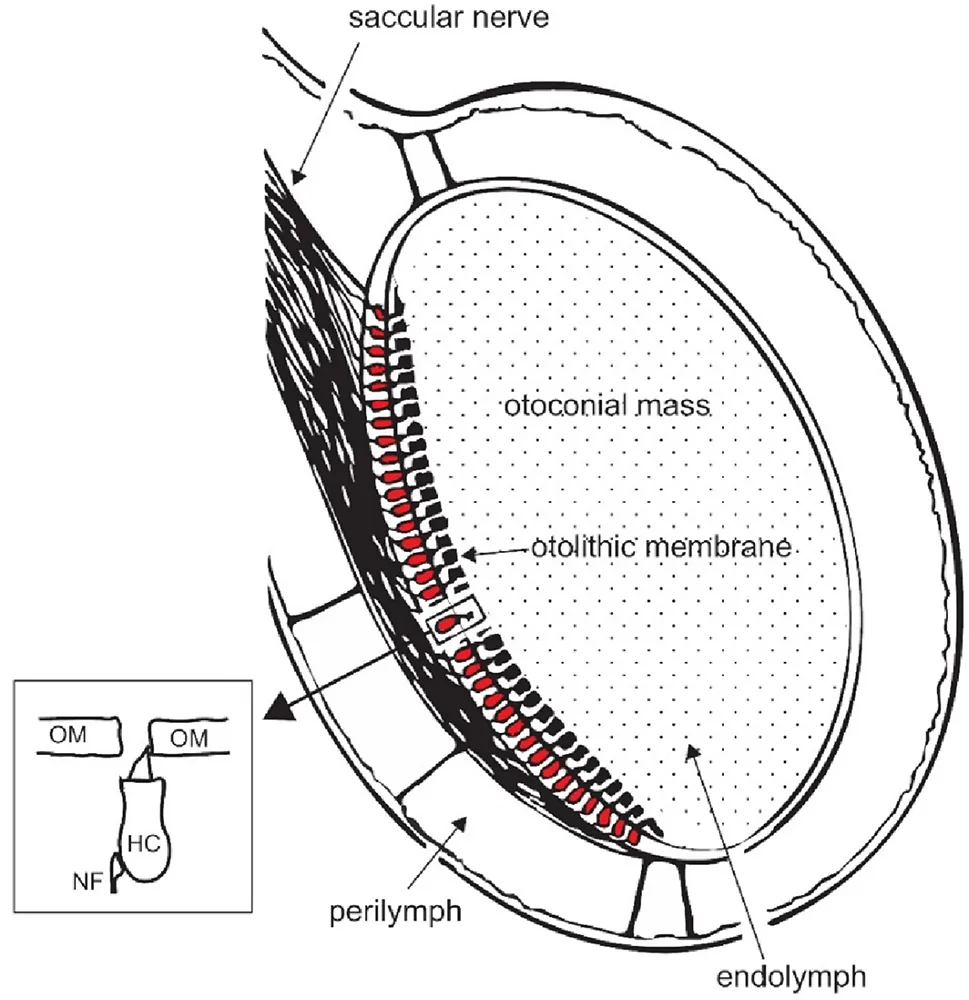
Schematic cross-section of the frog sacculus. The orientation of the macula is almost vertical, and the kinocilia of the hair cells (red) are coupled to the otolithic membrane through pores in the membrane (insert). HC, hair cell; NF, nerve fibre; OM, otolithic membrane. Redrawn from Eatock, R.A., Corey, D.P. & Hudspeth, A.J. (1987) Adaptation of mechanoelectrical transduction in hair cells of the bullfrog’s sacculus. *Journal of Neuroscience* 7: 2821-36. Copyright 1987, Society for Neuroscience

The saccular and lagenar otoconia form a suspension which is contained within the thin membranes that divide the endolymph and perilymph (Lewis and Narins 1999). Mechanical damage to these membranes, which readily occurs during dissection, results in the disintegration of the otoconial mass into a milky slurry (Marmo et al. 1983a; Lewis and Narins 1999; pers. obs.). Therefore, although we shall for convenience refer to the otoconial masses in frogs as ‘otoliths’, it is important to recognise that otoliths in frogs are not solid structures like those found in teleost fishes (Popper et al. 2005). The suspension of otoconial crystals within the amphibian sacculus is somehow attached to a gelatinous layer of matrix called the ‘otolithic membrane’. A fibrous layer interposed between the otolithic membrane and the surface of the sensory macula helps to hold the membrane in place (Hillman 1976; Kachar et al. 1990). The otolithic membrane is connected to the kinocilia of hair cells that protrude through pores in the membrane (Fig. 2), thus coupling the movements of the otoconial mass to the hair cells within the macula (Eatock et al. 1987; Kachar et al. 1990; Fig. 2). As in other vertebrates, the hair bundles in frogs assume an opposite polarity on either side of a ‘line of polarity reversal’, within the sensory maculae of both the sacculus and utriculus (Lewis and Li 1975; Harada et al. 2002). In mice, afferent neurons from each hemi-macula project to different central targets (Maklad et al. 2010), and the correct hair bundle polarity is required for normal vestibular function (Ji et al. 2022). The functional implications of the polarity reversal in frogs remain unknown.

The frog sacculus was initially thought to be an organ of equilibrium like the utriculus, until McNally and Tait (1925) showed no deficiencies in head orientation after ablation. Later, Ashcroft and Hallpike (1934) recorded from the cut saccular nerve in the opened otic capsule and measured responses to substrate vibrations. They showed that the saccular nerve responded solely to vibrations of the platform (and not to tilt and rotation), either generated by light objects falling on the platform, or by tuning forks tuned to 256–1024 Hz.

Whereas these studies conclusively demonstrated that the anuran sacculus sensed substrate vibrations, its sensitivity was only quantified nearly 50 years later in intact frogs. In an impressive study of vibration-sensitive neurons in the auditory nerve of the bullfrog *Lithobates catesbeianus* (formerly *Rana catesbeiana*), Koyama et al. (1982) found 48 single units that responded to low-frequency vibration stimuli with phase-locked responses down to accelerations of 0.005 cm s^− 2^. Based on earlier tracing experiments, these vibration-sensitive units were thought to innervate the sacculus or lagena. Later experiments on the white-lipped frog *Leptodactylus albilabris* demonstrated increased sensitivity, with phase-locked responses to stimuli below 0.001 cm s^− 2^ (Narins and Lewis 1984). These results revealed that frogs are among the most vibration-sensitive terrestrial vertebrates. Sensitivity to low-frequency vibrations (10–100 Hz) was largest, and the responses were thought to originate in the sacculus. This was later confirmed in the European grassfrog *Rana temporaria*, where recordings directly from the saccular nerve showed that almost all saccular fibres could be stimulated by vibrations, with comparable sensitivity to that found in the earlier studies (Christensen-Dalsgaard and Jørgensen 1988). Further work on the Northern leopard frog *Lithobates* (formerly *Rana*) *pipiens* (Christensen-Dalsgaard and Narins 1993) suggested that exquisite vibration sensitivity might be a general feature of anurans. Salamanders seem to have a lesser sensitivity to seismic vibrations (Capshaw et al. 2020).

Concurrently, frog saccular hair cells were established as a model system for the understanding of hair cell transduction (Eatock et al. 1987), because the hair cells could be isolated from the saccular macula and stimulated *in vitro.* Research on saccular hair cells led to a simple mechanical model (gating spring model) for hair cell mechanoreception (Hudspeth et al. 2000), and ultimately to the characterization of the mechanoreceptor channel.

#### Responses of saccular afferent fibres

As mentioned above, saccular afferent neurons in several species of frogs have been found to be highly sensitive to low-frequency vibrations of under a few hundred Hertz (Koyama et al. 1982; Lewis et al. 1982; Narins and Lewis 1984; Christensen-Dalsgaard and Jørgensen 1988; Christensen-Dalsgaard and Narins 1993), with similar sensitivity in all species studied. Low-frequency airborne sound can also stimulate these fibres, perhaps through its effects in eliciting skull vibration (Lewis et al. 1982; Yu et al. 1991; Christensen-Dalsgaard and Narins 1993). The frequency range of sound sensitivity in the saccular fibres overlaps the range of the low-frequency hearing organ, the amphibian papilla, but the sound sensitivity is generally lower for the saccular fibres (Christensen-Dalsgaard and Narins 1993).

**Fig. 3:**
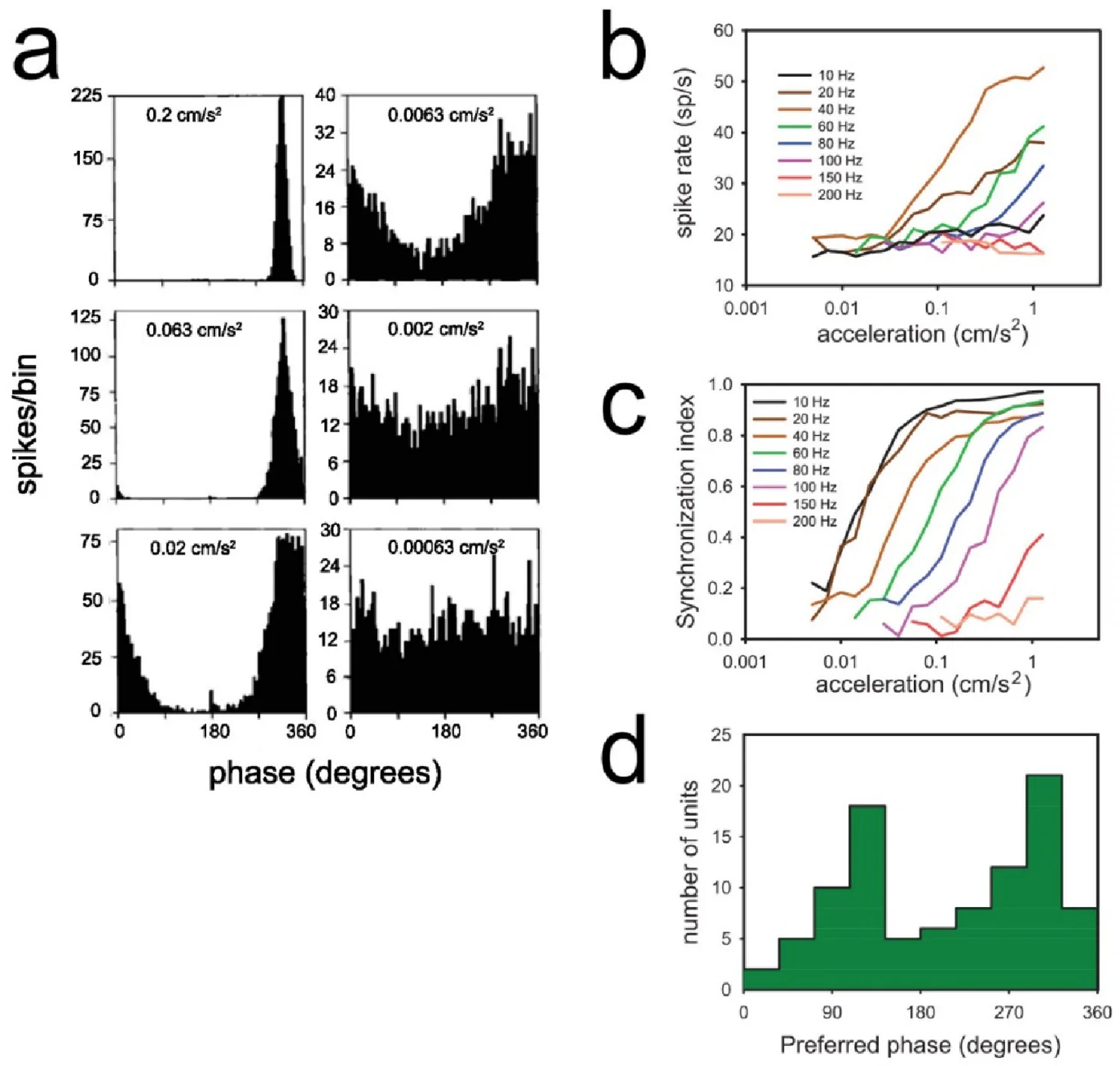
**a**: Responses of a vibration sensitive VIIIth nerve fibre in *Leptodactylus albilabris* to substrate vibrations. The figures are phase histograms showing the phase locking to vibration stimuli at different acceleration amplitudes at 130 Hz, bin width 3.6 degrees. **b** & **c**: Spike rate response **b** and synchronization responses **c** of a saccular fibre in *Rana temporaria* stimulated at frequencies from 10–200 Hz. d: Histogram of preferred phases of 95 single fibres in *R. temporaria*. **a** reprinted with permission from Narins, P.M. & Lewis, E.R. (1984) The vertebrate ear as an exquisite seismic sensor. *Journal of the Acoustical Society of America* 76: 1384–1387. Copyright 1984, Acoustical Society of America. **b**-**d** redrawn from Christensen-Dalsgaard, J. & Jørgensen, J.M. (1988) The response characteristics of vibration-sensitive saccular fibers in the grassfrog, *Rana temporaria*. *Journal of Comparative Physiology A* 162: 633–638, Springer Nature, reproduced with permission from SNCSC

An example of the response of a saccular afferent fibre to substrate vibrations is shown by the phase histograms in Fig. 3a (from Narins and Lewis 1984). The response to low-level vibrations (below approximately 0.01 cm s^− 2^ in this example) consists of phase-locking to the stimulus waveform, without any increase in average spike rate above spontaneous rate. At higher stimulus levels, average spike rate increases, usually to a limit of 1 spike/stimulus cycle for sinusoidal stimulation, and the phase locking increases with level until nearly all spikes fall within few phase degrees (Christensen-Dalsgaard and Jørgensen 1988). Figure 3b shows the spike rate response of a representative fibre to vibrations from 10 to 200 Hz, and Fig. 3c shows the phase locking response of a fibre quantified by the synchronization index, the normalized mean vector amplitude calculated from phase histograms such as the histograms in Fig. 3a. A synchronization index of 0 is an even distribution of spikes, i.e. no phase locking, while an index of 1 indicates that all spikes are concentrated in one bin. A comparison of the synchronization and spike rate responses clearly shows that the phase locking response is found at lower stimulus amplitudes than the spike rate response. Up to a synchronization index of 0.5, the modulation amplitude of neural firing is proportional to stimulus amplitude (i.e., the response is linear). Stimulus levels resulting in synchronization indices above 0.5 produce an increasingly nonlinear response and increase the average spike rate. Since the phase-locking response is just a redistribution of spontaneous activity it does not have a clear threshold, and the phase locking is probably only limited by masking by Brownian motion or physiological noise, for example generated by blood flow and arterial pulsations (Christensen-Dalsgaard and Jørgensen 1988).

From the phase-locked response, the preferred phase can be calculated. For a sample of 95 fibres, the distribution of preferred phases is clearly bimodal with two peaks 180 degrees apart. These two peaks are the expected response if the otolith were subject to linear translation in response to the stimulus. However, the occurrence of intermediate preferred phase values in some fibres is an indication that the movement of the saccular otolith is not a simple linear translation (Christensen-Dalsgaard and Jørgensen 1988; Benser et al. 1993; Kondrachuk 2000).

#### Responses of afferent fibres from the lagena and utricle

Recordings from bullfrogs showed that the central part of the lagenar macula is sensitive to low-frequency substrate vibrations, while the outer part responds to linear accelerations such as gravitational acceleration (Lewis et al. 1982; Baird and Lewis 1986; Cortopassi and Lewis 1998). Lagenar afferent responses have slightly broader pass-bands than saccular equivalents, suggesting that the lagenar fibres might have better temporal resolution and saccular fibres better frequency resolution (Cortopassi and Lewis 1998). However, in a recording from the lagenar nerve in *Rana temporaria*, no vibration sensitive fibres were found (sample of 57 fibres: Jørgensen and Christensen-Dalsgaard 1991). The lagenar nerve projections, mostly to vestibular nuclei in the central nervous system, suggest that the organ is primarily an organ of equilibrium (maybe sensitive to changes in head position: Khorevin 2008), and generally contributes little to vibration sensitivity.

Utricular afferent fibres in frogs are sensitive to head orientation with respect to the gravitational vector, or changes thereof, but they appear not to be vibration-sensitive (Lewis et al. 1982; Baird and Lewis 1986; Christensen-Dalsgaard and Narins 1993). The utriculus therefore seems to be exclusively an organ of equilibrium in frogs, but less is known about it than about either the sacculus or lagena.

#### The sacculus as an inertial motion sensor

At their simplest, the otoliths of frogs can be regarded as lumped masses connected to the sensory maculae through viscoelastic structures. The density difference between the otoconia and the inner ear fluids causes a time lag between the otoliths and the rest of the head, when the head is vibrated. This produces a shearing force that bends the stereocilia of the hair cells. As such, these organs should be capable of acting as inertial motion sensors (Lewis 1984; Cortopassi and Lewis 1998; Grant and Curthoys 2017).

The simplest model of otolith movement is as a harmonic oscillator with elasticity and damping. Such a model was proposed by de Vries (1950) for fish. Depending on the damping force, the otolith can be underdamped, with a prominent resonance peak, critically damped or overdamped. A theoretical analysis of fish otoliths suggests that a larger otolithic mass increases sensitivity (i.e. increased amplitude of movement of the otolith relative to its sensory macula, in response to head vibrations), while the peak response would shift to lower frequencies and become sharper (Lychakov and Rebane 2000). This assumes an otolith density greater than that of endolymph. The model of otolithic vibrations developed by Grant and Curthoys (2017), based on data from turtles and mammals, produces similar results to the fish models cited above. Grant & Curthoys note that otoliths work as seismometers above their undamped natural frequencies, and accelerometers below. Therefore, a larger otolithic mass, which decreases the natural frequency, would expand the working range as a seismometer. Using a more sophisticated model which takes into account otolith dimensions, Kondrachuk (2003) concluded that otolith thickness, rather than mass, is the relevant parameter in determining sensitivity, along with properties of the gel layer surrounding the hair cell bundles. However, Kondrachuk was considering an otolith operating below its natural frequency, within the accelerometer range.

The motion within the frog sacculus is likely much more complicated than such models suggest, since the otoconial mass is presumably deformable, unlike the solid fish otoliths, the hair bundle stiffness can vary dynamically (Benser et al. 1993) and the gel layer underneath appears inhomogeneous (Kondrachuk 2000). Although the saccular macula is oriented in the vertical plane, the crystalline otoconial mass remains articulated with the hair bundles, evidently held in place at least in part by the thin epithelial walls that surround it (Lewis and Narins 1999). The gain tuning curves from saccular afferents in *Lithobates* are very steep-sided, showing that the overall process of signal transduction from vibration to saccular afferent response is indeed much more complex than predicted from a simple mass-spring model (Lewis 1988). However, the general relationship between otolith size, natural frequency and sensitivity to head vibrations is expected to hold: a larger otolith, with greater mass and thickness, should augment seismic sensitivity.

#### Exteroception using the otolithic organs

In mammals, the utriculus and sacculus are primarily involved in detecting linear accelerations associated with body movements, but the vibratory sensitivity found in the frog sacculus suggests that it may have a role in exteroception, i.e. the detection of sensory cues arising outside the body. It is important to note that the function of an otolithic organ is largely defined by the orientation of its macula (sensory epithelium). The utricular macula is approximately horizontal, responding best to horizontal acceleration and tilting. In contrast, the saccular macula is vertical, making the sacculus most sensitive to vertical vibrations, notably including substrate vibrations. The frequency range of saccular fibres, generally 10–100 Hz, is optimal for detection of the most prominent type of substrate vibrations, Rayleigh waves, which propagate in the substrate surface (Narins 1990). Rayleigh waves are relatively slow seismic waves with velocities around 100 m s^− 1^, depending on soil properties.

The most general use of vibration sensitivity in frogs may be detection of seismic cues produced by approaching predators (Christensen-Dalsgaard and Jørgensen 1988; Lewis et al. 2001). However, some species probably communicate using vibration signals. Male *Leptodactylus albilabris* frogs calling on muddy substrates produce seismic vibrations concomitant with their sound emission, when their gular pouch hits the substrate; it was suggested that these vibrations are detected via the sacculus (Lewis and Narins 1985; Narins 1990).

Being routinely exposed to ground vibrations, it is to be expected that the seismic communication channel is used for a range of purposes by subterranean animals (Mason and Narins 2001). Saccular otoliths are very large in burrowing tortoises (Bramble 1982) and snakes (Olori 2010; Yi and Norell 2015), while vestibular inflation suggestive of saccular otolithic expansion has been found in caecilians (Maddin and Sherratt 2014) and a fossilised burrowing synapsid (Laaß 2015). Massively enlarged middle ear ossicles in some burrowing mammals are proposed to subserve a form of seismic sensitivity (Rose and Emry 1983; Mason 2003a, b, 2006). Some frogs from arid regions aestivate during dry seasons in subterranean burrows, emerging only when it rains. It has been shown that ground vibrations associated with rainfall can be used as cues for emergence (Dimmitt and Ruibal 1980; Márquez et al. 2016), and Márquez et al. suggested that the sacculus, along with the amphibian papilla, might be responsible for mediating this response. Might fossorial frogs have particularly large saccular otoliths, convergent with what is found in other burrowing vertebrates?

Although our focus so far has been on the detection of ground vibrations, fish otoliths are used in the detection of underwater sound; they can be stimulated directly, or indirectly via pulsations of the swim-bladder (Rogers et al. 1988; Popper and Lu 2000). Otolith morphology has been related to the particular aquatic habitat and lifestyle of fishes (see Schulz-Mirbach et al. 2019 for a review), and this has been extended to amphibian tadpoles (Bassó et al. 2016). Hypothetically, the otolith organs could be involved in transducing vibrations produced directly by particle motion of underwater sound waves, but this is unlikely. Underwater sound propagation, especially in shallow-water ponds where frogs are typically found, is restricted to higher frequencies to which the otolith organs are very insensitive (Christensen-Dalsgaard and Elepfandt 1995) and where the vibrations of the inner-ear fluids produced by the tympanic middle ear will be much larger than the particle motion generated by sound (Christensen-Dalsgaard et al. 1990). The cutoff frequency, below which sound does not propagate, depends on water depth and bottom substrate material. For example, in a shallow pond with sandy bottom and depth of 1 m, the cutoff frequency will be approximately 1 kHz, declining to 700 Hz at 2 m depth (Rogers and Cox 1988; Christensen-Dalsgaard 2025). However, surface waves produced by frogs in breeding ponds might, at least in principle, be detected by conspecifics using the otolithic organs of the inner ear (Christensen-Dalsgaard and Jørgensen 1988).

### Part 2: otolith size and frog lifestyle

If the sacculus in adult frogs is indeed involved in seismic vibration detection, species that would benefit most from these abilities might be expected to have the largest saccular otoliths. By analogy with other vertebrates, this might include fossorial species. We took the opportunity to test this hypothesis by comparing the volumes of the saccular otoliths in 11 species of African frogs which we had CT-scanned ourselves, as part of previous studies. These included the Arabian toad *Sclerophrys arabica* which is believed to aestivate underground, six *Leptopelis* species, all of similar skull size but with lifestyles ranging from semi-fossorial to fully arboreal, and other species which are considered amphibious, terrestrial or arboreal. In order to extend our survey to more highly specialised, burrowing frogs, we also looked at CT scans of three such species from the MorphoSource repository (www.morphosource.org): the Mexican burrowing toad (*Rhinophrynus dorsalis*; Rhinophrynidae), the Indian purple frog (*Nasikabatrachus sahyadrensis*; Nasikabatrachidae) and the Guinea snout-burrower (*Hemisus guineensis*; Hemisotidae). Finally, we considered an aquatic specialist, the African clawed frog (*Xenopus laevis*).

## Material and methods

Micro-CT scans were made from 15 adult specimens of 11 African frog species. *Leptopelis* specimens (Arthroleptidae) included *Leptopelis diffidens* (*n* = 2), *L. gramineus* (*n* = 1), *L. rugosus* (*n* = 2), *L. shebellensis* (*n* = 1), *L. susanae* (*n* = 1) and *L. xeniae* (*n* = 1). In addition to these, we examined *Afrixalus clarkei* (Hyperoliidae; *n* = 2), *Hyperolius viridiflavus* (Hyperoliidae; *n* = 1), *Phrynobatrachus* cf. *inexpectatus* (Phrynobatrachidae, *n* = 1), *Ptychadena delphina* (Ptychadenidae; *n* = 1) and *Sclerophrys arabica* (Bufonidae; *n* = 2). These specimens were all collected by one of the authors (SG) for use in other studies and had been sampled according to permits DA31/305/05, DA5/442/13, DA31/454/07, DA31/192/2010, DA31/230/2010, DA31/7/2011 and DA31/02/11 issued by the Ethiopian Wildlife Conservation Authority and the Fujairah Environment Authority. Specimen numbers, sex and measurements are presented in Supplementary Table 1. Individuals were euthanized by ventral application of 20% benzocaine gel. This sampling method was approved by the relevant Institutional Animal Care and Use Committee at Queens College and New York University School of Medicine (IACUC; Animal Welfare Assurance Number A32721–01 and laboratory animal protocol 19–0003). They were subsequently fixed in 10% unbuffered formalin for 24 to 48 h, and then transferred to 70% ethanol. All of these specimens, except for the *Sclerophrys*, were collected in 2018 and kept in ethanol until scanning in the summer of 2025. The *Sclerophrys* specimens were collected and fixed a few months before scanning. Before the scanning, specimens were rehydrated to 30% ethanol. They were scanned at NYUAD core facilities with a SKYSCAN 1276 micro-CT scanner (Bruker, Kontich, Belgium) at 45 kV and 200 µA, with a 0.25 mm aluminum filter. Scans were acquired with 450 projections and an exposure time of 377 ms per projection. Cubic voxel side-lengths for these scans were 37.35 μm.

The heads of 4 male African clawed frogs (*Xenopus laevis*; Pipidae) had been CT scanned as part of a previous study. These animals came from a breeding colony at the University of Cambridge and had been euthanized in October 2018 by a blow to the head administered by a custom captive-bolt device for the purposes of an undergraduate practical class, a procedure approved by the University of Cambridge Animal Welfare and Ethical Review Body. These four specimens had been kept frozen at -20 °C for 8 months before scanning, but had not been chemically preserved. After defrosting, their heads were scanned in the Cambridge Biotomography Centre with a Metris X-Tek HMX 160 micro-CT scanner, using settings of 125 kV and 120 µA. The images were each constructed from 1080 projections, with 1000 ms exposure per frame and 2 frames averaged per projection. Cubic voxel side-lengths for these scans were 18.3 μm. We later obtained a further 6 female *Xenopus* which had been euthanized in the same way for a practical class in October 2025 and had been kept frozen for 6 months, plus 1 female from the same colony which had died naturally in April 2026 and had been frozen for just 9 days. After defrosting, these animals were scanned in an upgraded scanner using settings of 110 kV and 110 µA, with 354 ms exposure per frame, 1080 projections and 2 frames averaged per projection. Cubic voxel side-lengths were 19.1 μm.

We also made reconstructions from CT scans of three species of highly-adapted fossorial frogs found in the MorphoSource repository (www.morphosource.org). These were the Mexican burrowing toad (*Rhinophrynus dorsalis*; Rhinophrynidae; CAS: HERP:71766, doi:10.17602/M2/M48893) from the California Academy of Sciences, collected in 1919, the Indian purple frog (*Nasikabatrachus sahyadrensis*; Nasikabatrachidae: CES-F-203, doi:10.17602/M2/M19512), from the Centre for Ecological Sciences, Bangalore, India, scan funded by the University of Florida, collection date unknown, and two specimens of the Guinea snout-burrower (*Hemisus guineensis*; Hemisotidae). One *Hemisus* was from the Louisiana State Museum of Natural History, collected in 2003 (LSUMZ: HERPS:87003, ark:/87602/m4/M100036), while the other was from the California Academy of Sciences, collected in 2015 (CAS: HERP:258533, doi:10.17602/M2/M34356), scan funded by the University of Florida. The *Rhinophrynus* and *Hemisus* scans formed part of the ‘Frog Diversity’ project run by the Blackburn Lab at the University of Florida’s Florida Museum of Natural History, managed by David Blackburn and Edward Stanley; the *Nasikabatrachus* scan formed part of the ‘Frogs of the Western Ghats, Peninsular India’ project, managed by David Blackburn. All these specimens appear to have been preserved in ethanol. The lifestyle of each species considered in this study is summarised in Table 1. Cubic voxel side-lengths for these MorphoSource scans were 29–48 μm.

**Table 1:** Lifestyles of the frog species considered in this study

Species	Lifestyle	References
*Afrixalus clarkei*	Arboreal, based on published photographs	Mertens et al. (2016)
*Hyperolius viridiflavus*	Terrestrial/arboreal, aestivating on dry vegetation and returning to water only to breed	Schmuck and Linsenmair (1997)
*Leptopelis* species	Variety of lifestyles, described as ranging from semi-fossorial (*L. rugosus*,* L. shebellensis*) to fully arboreal (*L. susanae*)	Goutte et al. (2022)
*Phrynobatrachus* cf. *inexpectatus*, *Ptychadena delphina*	Amphibious. Typically found on the ground; males call from the water of ponds or puddles	Largen (2001); Goutte et al. (2021)
*Sclerophrys arabica*	Typically found near permanent water sources but said to be able to aestivate underground, possibly for years	Balletto et al. (1985); Soorae et al. (2013)
*Hemisus guineensis*, *Nasikabatrachus sahyadrensis*,* Rhinophrynus dorsalis*	Highly fossorial, spending much of their lives underground	Emerson (1976); Trueb and Gans (1983); Senevirathne et al. (2016)
*Xenopus laevis*	Generally considered fully aquatic, although overland movement is documented	Measey (2016)

The tomogram stacks were converted to jpg or png format and opened in Stradview 7.321, written by Graham Treece & Andrew Gee of the Department of Engineering at the University of Cambridge (Treece et al. 1999). Stradview was used to make 3D reconstructions of the skulls and saccular otoliths and, if visible, the utricular and lagenar otoliths too. Reconstructions depended upon the choice of a particular greyscale threshold, such that all voxels brighter than that threshold were interpreted as either bone or otolith, and all those darker were ignored. The choice of threshold in each case was made subjectively by the same member of the author team with the aim of creating the most accurate reconstructions, based on close visual inspection of the tomograms. Where the automated sectioning procedure based on this threshold had made an obvious error, for example merging skull and adjacent otolith, the boundaries were manually adjusted. The volumes of all visible otoliths on both sides of the head, established using the same threshold, were recorded. Reconstructions were laterally inverted where necessary, to facilitate comparison.

Since cranial width is a commonly-used index of body size in frogs, we measured maximum skull width from the Stradview reconstructions: depending on skull morphology, the exact anatomical points that were measured between differed according to species. Because skull proportions varied, skull volumes were also taken from Stradview reconstructions as a second index of size. Measured skull volumes included mandibles but excluded otoliths, columellae and otic opercula. Otolith volumes were averages of those on the left and right sides of each specimen.

For the regression analyses, where data were available from more than one specimen of the same species, those data were averaged to yield one point per species. We then performed an ordinary least-squares (OLS) regression on the data. To take into account the relationships between the frog species considered, we also fitted a phylogenetic generalized least-squares (PGLS) regression line to the logged data using the *gls* function from the R package *nlme* (Pinheiro et al. 2013). To account for phylogenetic non-independence across the species, we built a covariance matrix using the *corBrownian* function from *ape* (Paradis and Schliep 2019) and a phylogenetic tree obtained by combining the time-calibrated phylogeny from Portik et al. (2023), which contains most anuran species, with the dated Ethiopian *Leptopelis* phylogeny from Reyes-Velasco et al. (2018). Male and female *Xenopus* were considered separately for the purposes of the regressions, using an artificial branch-length of zero for the PGLS regression.

For the purposes of assessing the effect of formalin solution on otolith volumes, we used the female *Xenopus* which had died in April 2026, one of the other female *Xenopus* from the October 2025 batch, plus another four female *Xenopus* from the same October batch, the mandibles of which had been removed and which therefore could not be included in the main study. The heads of these six specimens were CT-scanned as above and then their heads were immersed for 48 h in 10% unbuffered formalin, diluted from 37 to 41% formaldehyde solution stabilised with ca. 12% methanol (Fisher Scientific, Loughborough). Having been removed from the fixative, the heads were rescanned with identical settings. The saccular otoliths were reconstructed using the same thresholds, both before and after the formalin fixation, and their volumes were compared.

## Results

**Fig. 4:**
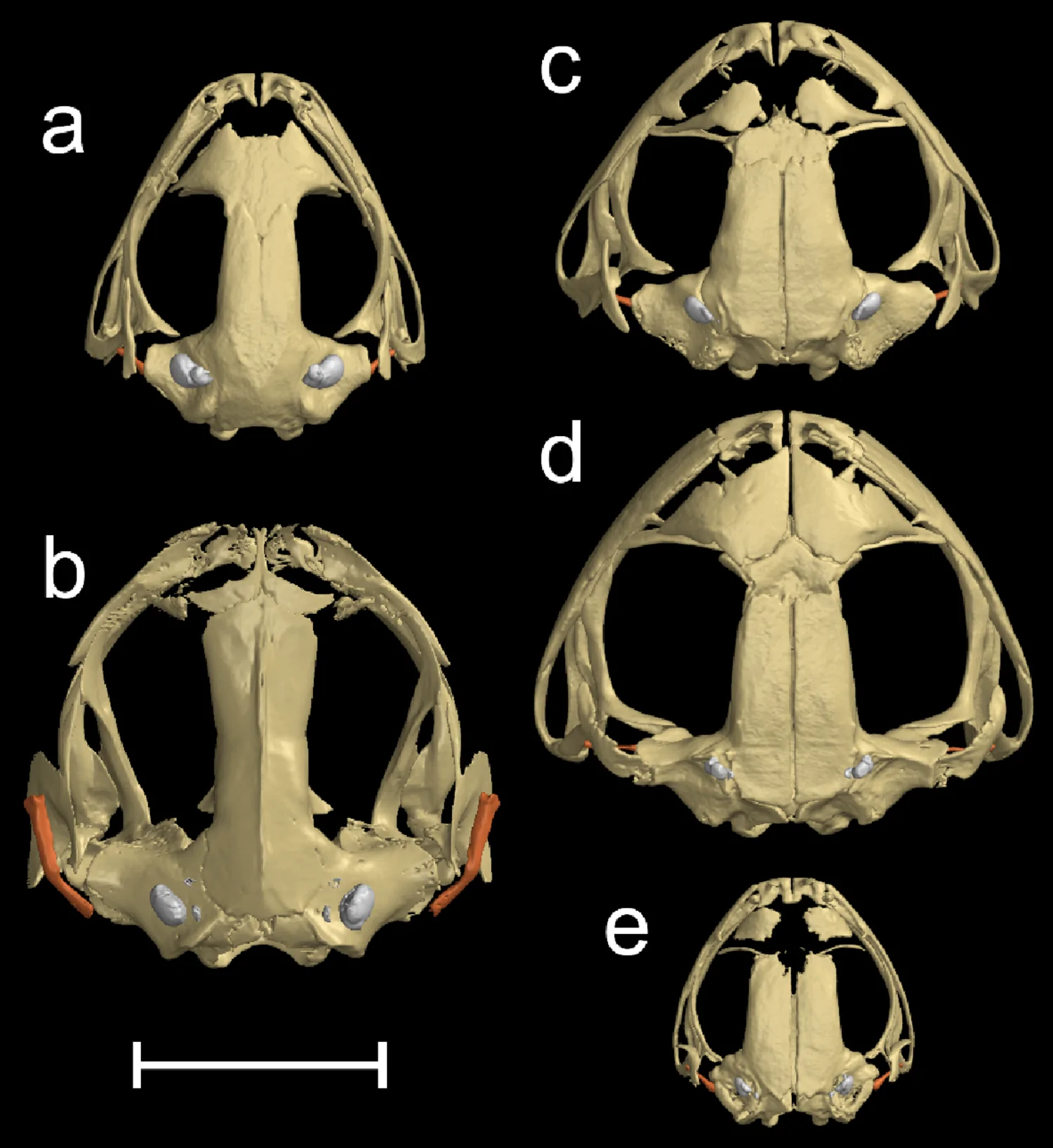
CT reconstructions of the skulls of five frog specimens. **a**: *Ptychadena delphina* SB310; **b**: *Xenopus laevis* XL14 (male); **c**: *Leptopelis rugosus* SB558; **d**: *Sclerophrys arabica* SB1328; **e**: *Hyperolius viridiflavus* SB180. Columellae are coloured orange; otoliths are shown in white and are superimposed onto the skulls. Scale bar 10 mm

**Fig. 5:**
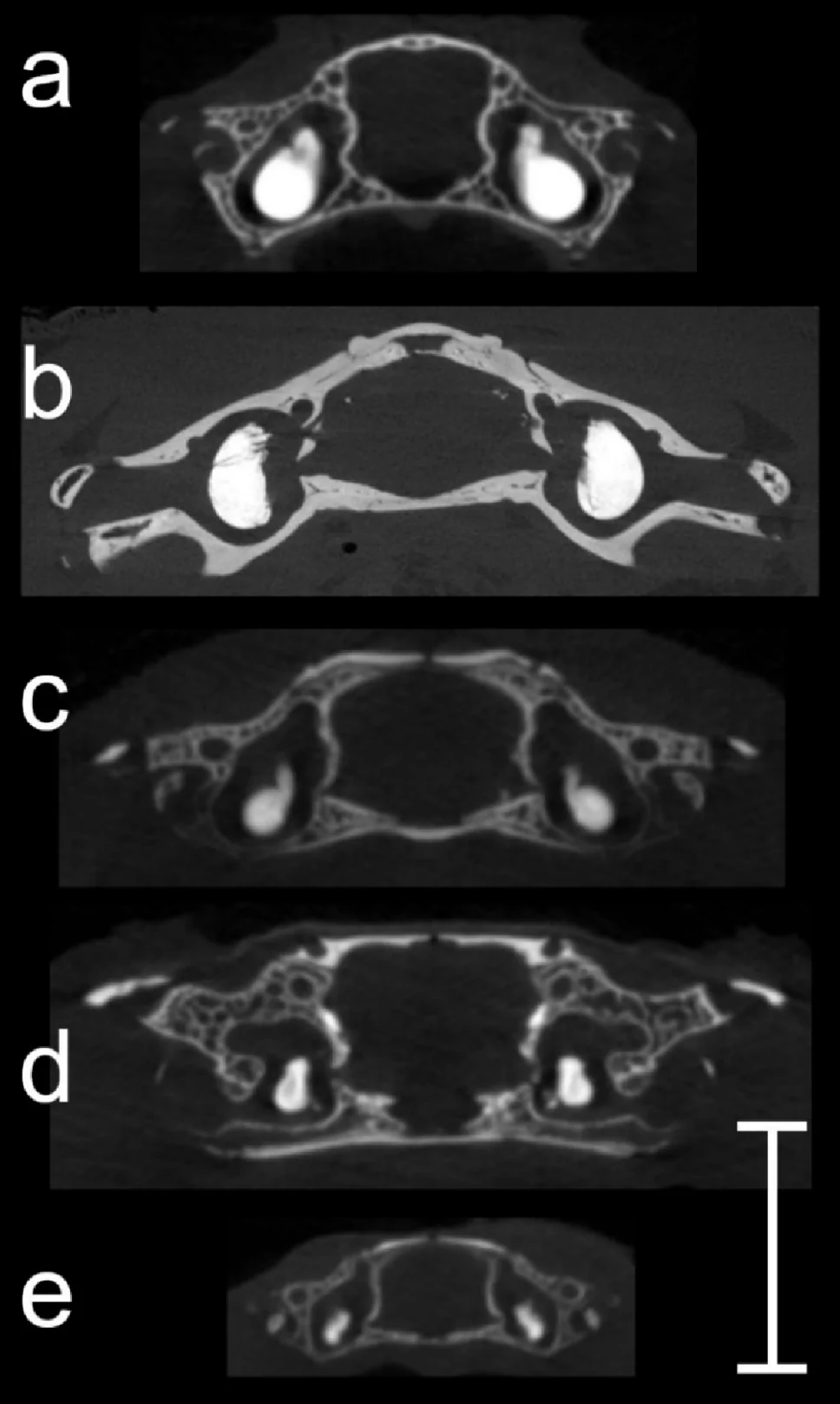
Transverse tomograms through the heads of five frog specimens. The sections were chosen to pass through the centres of the saccular otoliths, which are visible as the white, rounded structures on each side. **a**: *Ptychadena delphina* SB310; **b**: *Xenopus laevis* XL14 (male); **c**: *Leptopelis rugosus* SB558; **d**: *Sclerophrys arabica* SB1328; **e**: *Hyperolius viridiflavus* SB180. Scale bar 5 mm

**Fig. 6:**
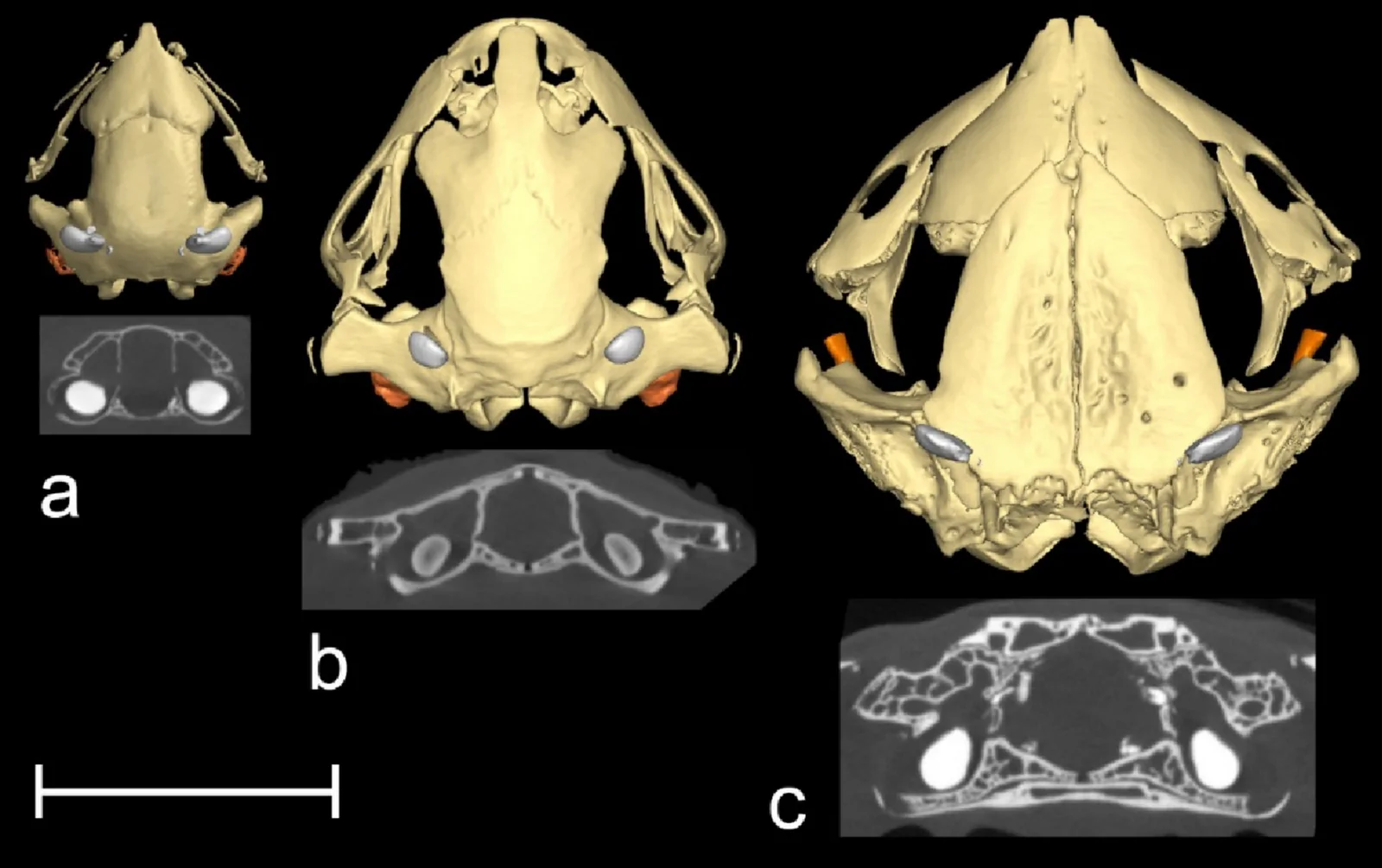
Reconstructions (above) and transverse tomograms (below) through the heads of three highly fossorial frog species. The tomogram sections were chosen to pass through the centres of the saccular otoliths. **a**: *Hemisus guineensis* (CAS: HERP:258533); **b**: *Rhinophrynus dorsalis* (CAS: HERP:71766); **c**: *Nasikabatrachus sahyadrensis* (CES-F-203). In the reconstructions, opercula (in **a** and **b**) and columellae (in **c**) are coloured orange. Otoliths, shown in white, are superimposed onto the skulls. Scale bar 10 mm

CT reconstructions and tomograms made from five representative specimens which we scanned ourselves are shown in Figs. 4 and 5, while Fig. 6 shows the same from the three highly fossorial species from MorphoSource. The middle ear structures of the frogs examined that were visible in the CT reconstructions are summarised in Table 2. Of the stapes (columella), only the bony shaft representing the *pars media* could be seen in the scans. Cartilaginous opercula could not be seen directly in the scans, but their presence was assumed based on the size of the opening into the bony otic capsule, posterior to the stapes.

**Table 2:** Middle ear structures in the frogs examined

Species	Middle ear structures
All *Leptopelis* species, *Afrixalus clarkei*,* Hyperolius viridiflavus*, *Nasikabatrachus sahyadrensis*,* Ptychadena delphina*,* Sclerophrys arabica*	Stapes and cartilaginous operculum
*Phrynobatrachus* cf. *inexpectatus*	Stapes and partly ossified operculum
*Hemisus guineensis*, *Rhinophrynus dorsalis*	Bony operculum only; possible vestigial stapes in *Rhinophrynus*
*Xenopus laevis*	Stapes only

**Fig. 7:**
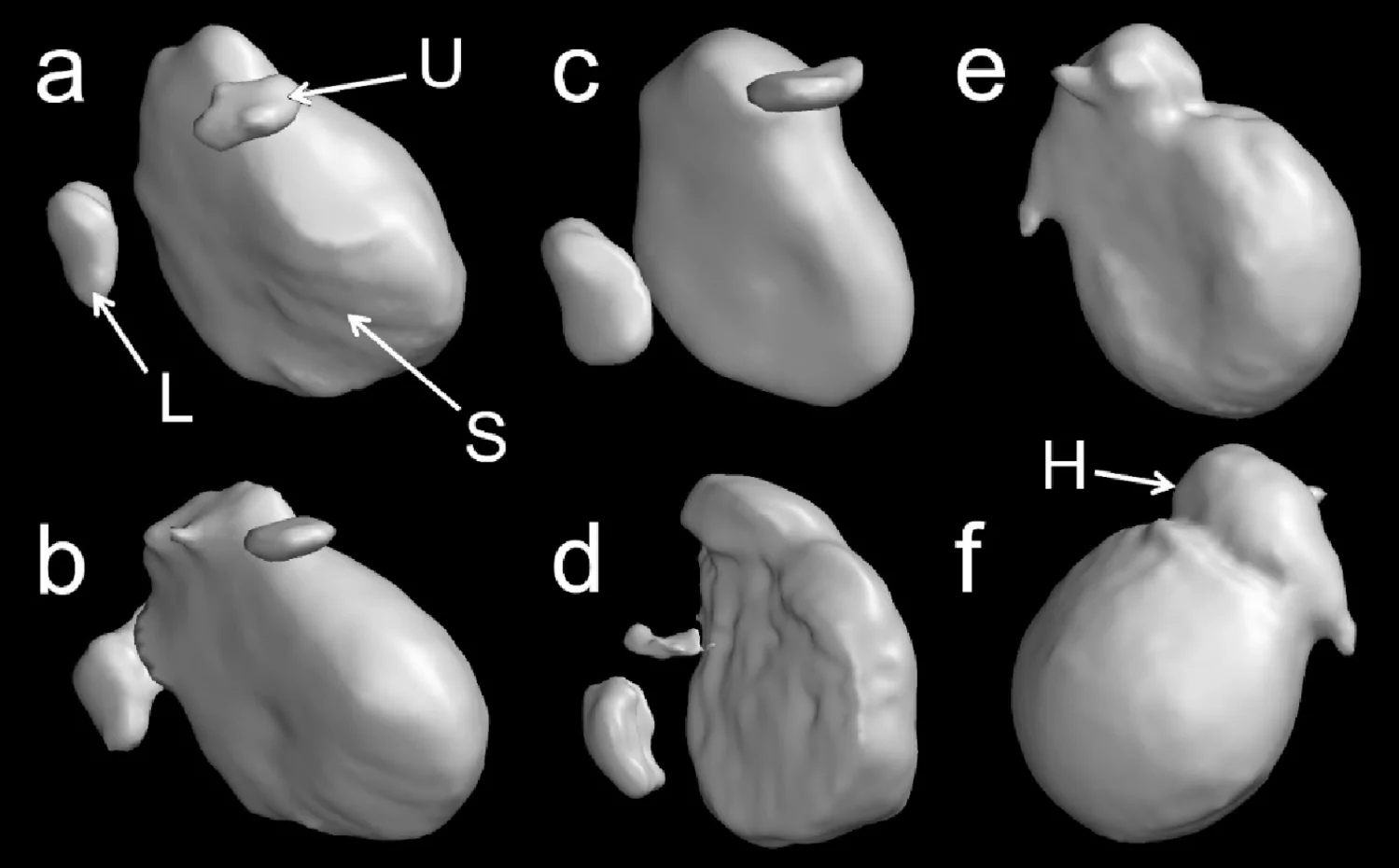
Reconstructions of the left otoliths of five species of frogs. **a**: *Leptopelis diffidens* SB110; **b**: *L. xeniae* SB188; **c**: *Sclerophrys arabica* SB1328; **d**: *Xenopus laevis* XL13 (male); **e**, **f**: *Ptychadena delphina* SB310. All shown from a rostral view except **f**, which is from a posterior view. The lagenar and utricular otoliths are not shown in the *Ptychadena* reconstructions; the narrow projections of the saccular otolith ‘head’ in this specimen might reflect dense soft tissue close to the otoconial mass. H = ‘head’ of saccular otolith; L = lagenar otolith; S = saccular otolith; U = utricular otolith. Not to scale

Saccular otoconial masses (‘otoliths’: Fig. 7) were lentil-shaped in *Rhinophrynus*, *Nasikabatrachus*, *Leptopelis shebellensis* and *Xenopus*, the flattened surface facing rostromedially and the longest axis approximately vertical. In the other species, there was a dorsomedial ‘head’ of this otolith, of variable degrees of prominence but most obvious when partially separated from the main part of the otolith by a groove on the posterolateral aspect (Fig. 7f).

**Fig. 8:**
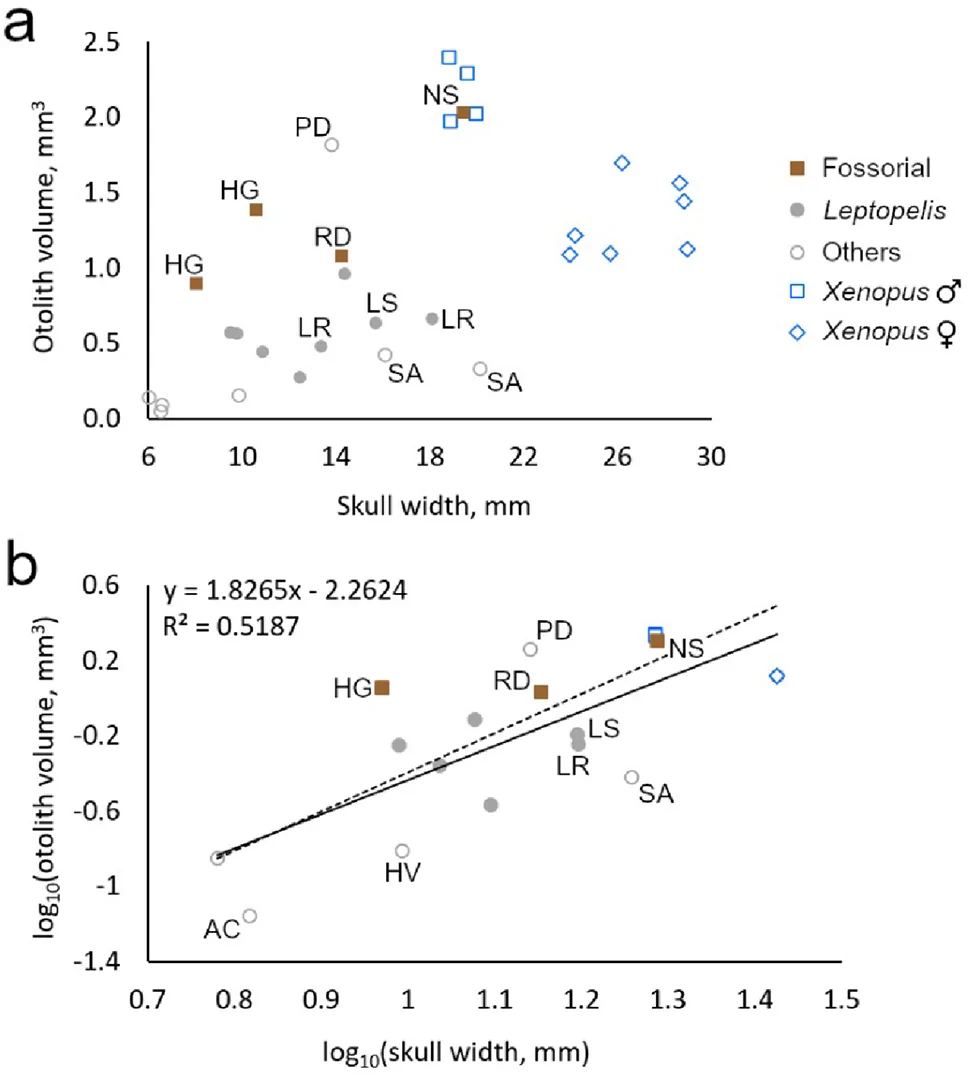
Relationship between the volumes of the saccular otoliths and maximum skull widths in the frog specimens examined. Otolith volumes are averages of left and right sides. **a**: regular axes; **b**: logarithmic axes. In **a**, each individual specimen is shown. In **b**, each species is represented by a single averaged point except for *Xenopus*, for which the average points representing males and females are shown separately. AC = *Afrixalus clarkei*; HG = *Hemisus guineensis*; HV = *Hyperolius viridiflavus*; LG = *Leptopelis rugosus*; LS = *Leptopelis shebellensis*; NS = *Nasikabatrachus sahyadrensis*; PD = *Ptychadena delphina*; RD = *Rhinophrynus dorsalis*; SA = *Sclerophrys arabica*. Solid line = OLS regression line; dashed line = PGLS regression line. The equation is for the OLS regression. Regression lines were calculated from all points shown in **b**

**Fig. 9:**
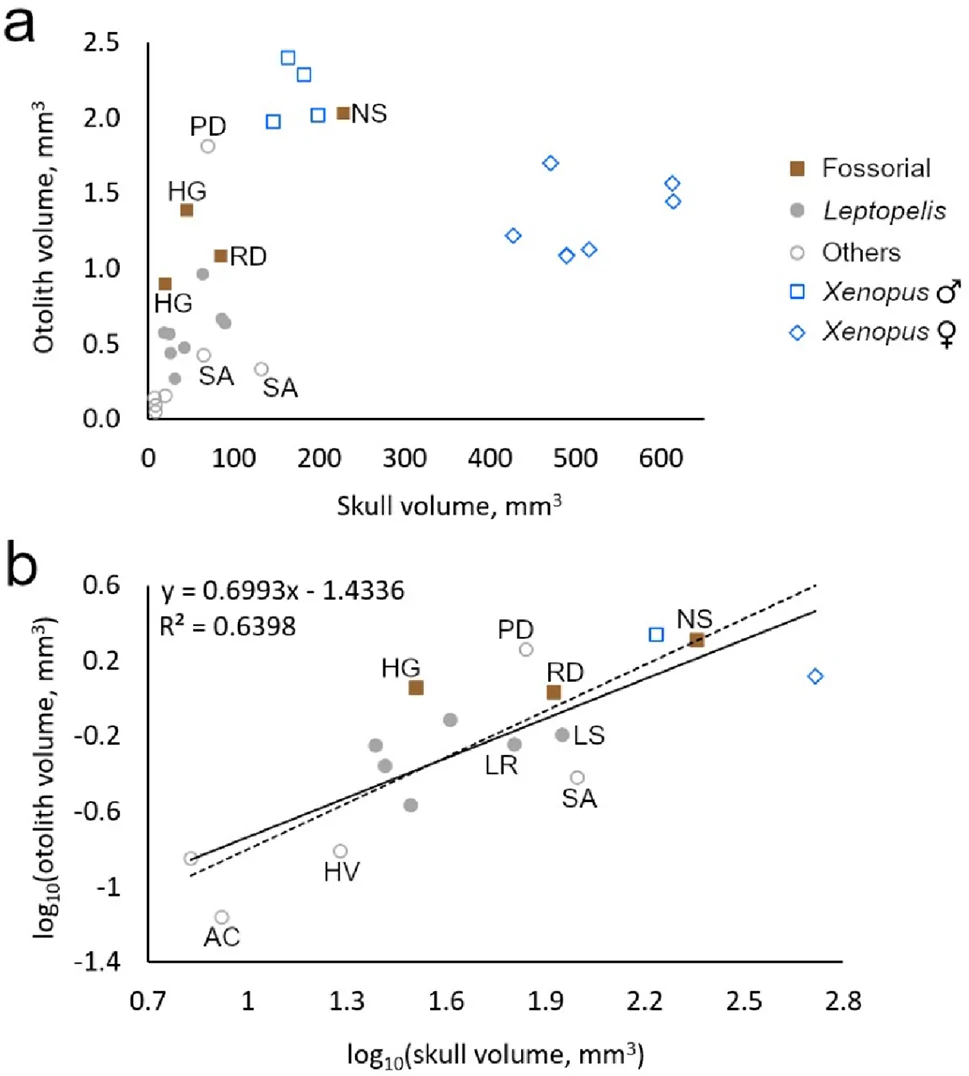
Relationship between the volumes of the saccular otoliths and skull volumes in the frog specimens examined. Otolith volumes are averages of left and right sides. Skull volumes include mandibles but exclude stapes, operculum and otoliths. **a**: regular axes; **b**: logarithmic axes. In **a**, each individual specimen is shown. In b, each species is represented by a single averaged point except for *Xenopus*, for which the average points representing males and females are shown separately. AC = *Afrixalus clarkei*; HG = *Hemisus guineensis*; HV = *Hyperolius viridiflavus*; LG = *Leptopelis rugosus*; LS = *Leptopelis shebellensis*; NS = *Nasikabatrachus sahyadrensis*; PD = *Ptychadena delphina*; RD = *Rhinophrynus dorsalis*; SA = *Sclerophrys arabica*. Solid line = OLS regression line; dashed line = PGLS regression line. The equation is for the OLS regression. Regression lines were calculated from all points shown in **b**

Male *Xenopus*, *Nasikabatrachus* and *Ptychadena* had the largest saccular otoliths in absolute terms (Figs. 8 and 9). Male *Xenopus* specimens and *Nasikabatrachus* clustered together. The saccular otoliths were smaller in volume in female *Xenopus* compared to those of males of the same species: using averaged volumes from left and right ears, all four male values were higher in absolute terms than any of the seven female values, and the difference was statistically significant (Mann-Whitney U-test, two-tailed, *p* = 0.006). This was particularly striking given that female *Xenopus* are substantially larger than males (Figs. 8 and 9).

In order to see which species had large otoliths relative to skull size, we calculated the *y*-axis residuals from the ordinary least squares (OLS) regression lines shown in Figs. 8b and 9b. *Hemisus* fell furthest above the lines and hence had relatively the largest otoliths, followed by *Ptychadena*. *Sclerophrys* fell furthest below the lines and hence had relatively the smallest otoliths, followed by *Afrixalus*. While male *Xenopus* had relatively large otoliths, those of female *Xenopus* were relatively small. In order to test the hypothesis that the three fossorial specialists, *Hemisus*, *Nasikabatrachus* and *Rhinophrynus*, collectively have larger otoliths than expected for their size, we compared the residuals for these three species with those for the 11 other species (excluding *Xenopus*), using a one-tailed Mann-Whitney U-test. There was a significant difference when considering the relationship with skull width (*p* = 0.019) but not skull volume (*p* = 0.063).

The phylogenetically-controlled (PGLS) regression lines had slightly steeper slopes than the OLS regressions but were similarly positioned (Figs. 8b and 9b). *Hemisus* fell furthest above these lines too, followed by *Ptychadena*. *Sclerophrys*, *Afrixalus*, *Hyperolius* and female *Xenopus* had relatively small otoliths.

The very small lagenar and utricular otoliths were much harder to discern than the saccular otoliths due to limited resolution: the best reconstructions were made from the specimens shown in Fig. 7a-d. The lagenar otolith, resembling a baseball glove in shape, concave laterally, was located posteromedial to the saccular otolith. Of the specimens shown in Fig. 7a-d, it was 3–4% of the saccular otolith volume in *Leptopelis* and *Xenopus*, 9% in *Sclerophrys*. The utricular otolith was the smallest, under 1.5% of saccular otolith volume in *Leptopelis* and *Xenopus*, 3% in *Sclerophrys*. It took the form of a flattened disc oriented in the horizontal plane, located anterior to the saccular otolith. The utricular otoliths of *Xenopus* were located further medially, relative to the other otoliths, than in the other species.

The volumes of saccular otoliths within six female *Xenopus* heads were compared, before and after 48 h of immersion in 10% formalin. Of the 12 otoliths, we calculated reduced volumes in 7 and increased volumes in 5, after fixation. Averaging left and right volumes from the same frog and comparing volumes before and after fixation, all differences were within 4% of original values. No significant differences were found between the otolith volumes before and after fixation (Wilcoxon signed-rank test: *p* = 0.813).

## Discussion

Otolith structure varies among different vertebrate groups. The evolution of these structures in diverse groups of fishes is considered in a comprehensive review by Schulz-Mirbach et al. (2019). Fish otoliths have been particularly well-studied since they are used for purposes including species identification and the assessment of aging and growth in fish populations (Campana 2005). They are solid structures in teleosts, so large and robust that they can even be retrieved from seal faeces (Pierce et al. 1991), and micro-computed tomography is a well-established method for their examination (Vasconcelos-Filho et al. 2019). Otoliths are conspicuous in CT scans of a range of reptiles (Olori 2010; Vasilopoulou-Kampitsi et al. 2019; Schwab et al. 2022), and are clearly visible but relatively thin in birds (Bonsmann et al. 2016; Malkemper et al. 2018). In contrast, otoliths are often completely invisible in CT scans of mammalian ears (Ekdale 2013) but otolithic material has been identified in high-resolution scans of specimens which had not been chemically preserved (Peacock et al. 2026). In all frog specimens investigated here, the saccular otoliths in particular were very prominent in CT scans. Otoliths must be denser than the surrounding fluids if they are to move relative to the sensory macula in response to head vibrations (Lychakov and Rebane 2005; Popper et al. 2005). Density was not assessed in this study in terms of mass per unit volume, but radiodensity of the saccular otoliths was higher than that of the surrounding skull in our frog specimens (Fig. 5), suggesting a high level of mineralization. This would be consistent with a high mechanical density.

Soft tissue structures could not be clearly seen in our CT scans but comparison with the reconstruction shown in Fig. 1, which was made from embedded serial sections, reveals that the saccular macula where the sensory hair-cells are located is spread over the flattened part of the saccular otolith. Most of the saccular otolith is contained within the inferior saccular chamber. Some species, including *Lithobates pipiens* (Mason et al. 2015) have a constriction separating inferior and superior saccular chambers. We interpret the ‘head’ of the saccular otolith, seen in species such as *Ptychadena* (Fig. 7f), as a relatively small component which projects into the superior chamber through this constriction. *Xenopus* lacks such a constriction (Mason et al. 2015), which is presumably why its saccular otolith is simpler in shape, without a ‘head’. Our interpretation differs from that of Lychakov (2004), who seemed to regard the ‘head’ as the base of the otolith, located below the main part and in contact with the sensory macula.

### Otoconial decalcification

Tadpoles possess saccular otoliths (Bassó et al. 2016). Otoconia in the endolymphatic sacs become demineralised during the process of metamorphosis, presumably to provide calcium to the developing skeleton, but the crystals in utricle, saccule and lagena are somehow protected (Pilkington and Simkiss 1966; Marmo et al. 1983b). Therefore, otoliths should in principle be identifiable even in very young frogs, and are clearly visible in CT scans of the earliest metamorphosed natterjack toadlets (*Epidalea calamita*) (Lauridsen and Christensen-Dalsgaard 2022).

The otoconia within the endolymphatic sacs of frogs are thought to be used as a buffer in vivo, dissolving in low pH conditions (Simkiss 1968), whereupon they can no longer be seen in x-rays (Sulze 1942). Unbuffered formalin solution can, over time, oxidise to formic acid (Howat and Wilson 2014): human calcite otoconia have been shown to be rapidly eroded in formalin, although their shape remains preserved via their organic matrix (Kniep et al. 2015). In our study, 48 h of immersion of *Xenopus* heads in unbuffered formalin was found to have very little effect on saccular otolith volume. The very small discrepancies in volume measured, some of which were increases, might simply have been due to noise in the scanning and reconstruction process. These otoliths were likely to have been protected to some extent from the limited exposure to formalin through their location within the otic capsule. Although Kniep et al. found that pure ethanol had no effect on human otoconia even after two years of exposure, ethanol solutions of under 80% can be acidic and result in otolithic dissolution, as documented in fishes (Butler 1992). Following initial preservation in formalin, many of our specimens had been transferred into ethanol solution for up to eight years, but the similarity in shape and size between right and left otoliths (Figs. 5 and 6), their similar volumes in different specimens of the same species (Figs. 8a and 9a), and their generally high radiodensity (Figs. 5 and 6) were all reassuring. This was also the case for the *Hemisus* and *Nasikabatrachus* specimens from MorphoSource. The saccular otoliths of the *Rhinophrynus* specimen, which had evidently been stored in ethanol for nearly a century before it was scanned, were less radiodense, especially centrally, but they were relatively large in volume and left and right otoliths were similar in shape (Fig. 6b). However, some other species had capacious inner ear cavities containing relatively small otoliths (Fig. 5c, d,e), so we cannot exclude the possibility that there may have been some shrinkage or dissolution. The possibility of preservative-induced changes in otolith volumes remains a significant concern which cannot be ignored.

### Otolith volume and ecology

Among land mammals, there is a close correlation (R^2^ = 0.90) between middle ear ossicle mass and skull mass, with the ossicles exhibiting negative allometry (Nummela 1995). Larger mammals have absolutely larger, but relatively smaller, ossicles. Constructing a regression line though data points representing a wide variety of species allows for the identification of outliers which have particularly large ossicles. We performed a similar analysis here, looking at the saccular otolithic volumes of frogs with respect to skull size, and also found negative allometry (Figs. 8b and 9b). Although saccular otolith volume generally increases with body size, our regression relationships reveal a lot of scatter. The correlations were looser than in Nummela’s (1995) middle ear study, with R^2^ = 0.52 and 0.64 for the OLS regressions on skull width and skull volume respectively. This might be a function of the skull size-range available to us being much narrower and/or the very diverse range of lifestyles among frogs in our sample.

Male *Xenopus* had the largest saccular otolith volumes in absolute terms, followed by *Nasikabatrachus* and *Ptychadena*, while *Hemisus* had the largest relative to skull size, followed by *Ptychadena*. The otoliths of *Hemisus* and *Ptychadena* were around twice the volume of those of the other species we examined of similar skull size. *Sclerophrys arabica* had the smallest saccular otoliths relative to skull size. The largest *Leptopelis* otoliths in absolute terms were found in a *Leptopelis diffidens* specimen, a species falling somewhere in the middle of the terrestrial to arboreal spectrum (Goutte et al. 2022), while *Leptopelis rugosus* and *L. shebellensis* both fell below the regression lines of Figs. 8b and 9b.

Although *Sclerophrys arabica* is suspected of burrowing and both *Leptopelis rugosus* and *L. shebellensis* are semi-fossorial (Table 1), none of these can be regarded as highly specialized towards fossoriality. Similar in general body shape to bufonid toads, they retain externally visible tympanic membranes. *Rhinophrynus*, *Nasikabatrachus* and *Hemisus*, however, show much more extreme morphological adaptations (Emerson 1976; Trueb and Gans 1983; Senevirathne et al. 2016), including highly unusual and robust skulls (Fig. 6). *Rhinophrynus* and *Hemisus* both lack functional stapedes (Table 2), suggestive of a lack of sensitivity to higher-frequency, airborne sound which would otherwise be transmitted to the inner ear through the tympanic route (Mason 2007). These bizarre frogs all have large saccular otoliths in absolute terms, and it is absolute rather than relative size which should relate to seismic sensitivity. However, only *Hemisus* stands out as having particularly large otoliths relative to skull size (Figs. 8 and 9). Among mammals, only some subterranean species have unusually large auditory ossicles believed to subserve seismic sensitivity, there being considerable variation even within members of the same family (Mason 2001, 2006). If the sacculus is used for the detection of ground vibrations in fossorial frogs, perhaps adaptive hypertrophy has occurred in a similarly patchy way.

Surprisingly, *Ptychadena delphina* had among the largest otoliths in both relative and absolute terms. This species comes from an African genus with “highly conserved morphological features” (Goutte et al. 2021): it closely resembles some of the ranid frogs such as *Lithobates pipiens* or *Rana temporaria*, familiar as laboratory species. Male *P. delphina* call from pools or puddles in flooded fields or roadways (Goutte et al. 2021). This is analogous to *Leptodactylus albilabris*, which Lewis and Narins (1985) found to use a seismic component in calling. This suggests that the use of substrate-borne vibrations in intraspecific communication could represent a significant selective pressure on otolith enlargement. However, the *Phrynobatrachus* species that we examined, which seems to have a similar lifestyle (Table 1), did not have enlarged otoliths. Further study of their reproductive behaviours may shed some light onto the reason for this difference.

An unexpected finding from this study was the pronounced sexual dimorphism in saccular otoconial volumes in male and female *Xenopus laevis*. Although male *Xenopus* are much smaller than females, males had significantly larger otoliths in absolute terms. Sexual dimorphism has previously been recorded in the middle ear of this aquatic frog, with males having relatively larger tympanic discs (Mason et al. 2009). The tympanic disc is a cartilaginous expansion of the extrastapes; pulsations of the lungs and middle ear air cavity underwater vibrate this disc, which in turn should result in the stapes footplate transmitting vibrations to the inner ear fluids, ultimately resulting in sound reception (Christensen-Dalsgaard et al. 1990; Christensen-Dalsgaard and Elepfandt 1995; Vedurmudi et al. 2018). It is believed that pulsations of the fish swim-bladder, passed to the inner ear, can result in vibrations of the saccular otolith, contributing to the perception of sound underwater (Schulz-Mirbach et al. 2019). Perhaps the saccular otoconial mass of *Xenopus* is used in a similar way, and its enlarged volume in males is functionally associated with the enlarged tympanic disc. It is notable that a study of auditory-evoked potentials has shown that male *Xenopus* are more sensitive to sound frequencies below around 1.5 kHz than are females (Hall et al. 2016).

Unfortunately, we were limited to the examination of just one or two specimens from each of the other species, so it remains uncertain whether they too exhibit sexual dimorphism in their otoliths. It would be particularly interesting to examine more *Nasikabatrachus* specimens, since the females in that species are also much larger than males (Zachariah et al. 2012).

In summary, we found considerable variation in saccular otolithic volumes between the frog species that we investigated. Although the most extreme fossorial specialists did have large otoliths in absolute terms, frogs with a lesser degree of fossoriality had relatively small otoliths. It must be recognised, however, that our data-set contains a disproportionate number of fossorial and aquatic specialists relative to anurans as a whole, which may have affected the position of our regression lines. The inclusion of just one or two individuals per species represents another clear limitation, given our identification of pronounced sexual dimorphism in *Xenopus*. This preliminary study should therefore be repeated with a more extensive data-set, but the problem of dissolution of otoliths in preservative solution over time complicates any wider comparison based on museum specimens. It is notable that the frogs so far studied experimentally have a comparable sensitivity to vibrations despite very different body sizes, from the relatively small *Leptodactylus albilabris* (Narins and Lewis 1984; Christensen-Dalsgaard and Narins 1993) to the much larger *Lithobates catesbeianus* (Koyama et al. 1982), but we lack measurements of vibratory sensitivity from fossorial frogs. There is thus a need for further studies of behaviour and physiology, continuing the landmark studies of Peter Narins.

## Supplementary Information

Below is the link to the electronic supplementary material.

Supplementary Material 1

## Data Availability

The CT data from the scans made at NYUAD are available on MorphoSource (www.morphosource.org, project ID# 000871171) while data from the *Xenopus* scans made in Cambridge are available on Zenodo (https://doi.org/10.5281/zenodo.20700652).
